# Effects of Rice-Husk Silica Liquid in Streptozotocin-Induced Diabetic Mice

**DOI:** 10.3390/metabo12100964

**Published:** 2022-10-12

**Authors:** Hsin-Yuan Chen, Yong-Han Hong, Yi-Fen Chiang, Kai-Lee Wang, Tsui-Chin Huang, Mohamed Ali, Tzong-Ming Shieh, Hsin-Yi Chang, Shih-Min Hsia

**Affiliations:** 1 School of Nutrition and Health Sciences, College of Nutrition, Taipei Medical University, Taipei 11031, Taiwan; 2 Graduate Programs of Nutrition Science, School of Life Science, National Taiwan Normal University, Taipei 106209, Taiwan; 3 Department of Nursing, Ching Kuo Institute of Management and Health, Keelung 20301, Taiwan; 4 Graduate Institute of Cancer Biology and Drug Discovery, College of Medical Science and Technology, Taipei Medical University, Taipei 11031, Taiwan; 5 Clinical Pharmacy Department, Faculty of Pharmacy, Ain Shams University, Cairo 11566, Egypt; 6 School of Dentistry, College of Dentistry, China Medical University, Taichung 40402, Taiwan; 7 Graduate Institute of Medical Science, National Defense Medical Center, Taipei 114, Taiwan; 8 Graduate Institute of Metabolism and Obesity Sciences, College of Nutrition, Taipei Medical University, Taipei 11031, Taiwan; 9 School of Food and Safety, Taipei Medical University, Taipei 11031, Taiwan; 10 Nutrition Research Center, Taipei Medical University Hospital, Taipei 11031, Taiwan; 11 TMU Research Center for Digestive Medicine, Taipei Medical University, Taipei 11031, Taiwan

**Keywords:** rice-husk silica liquid, food-grade silica liquid, rosiglitazone, streptozotocin, diabetic liver injury

## Abstract

Type 2 diabetes mellitus is a complex multifactorial disease characterized by poor glucose tolerance and insulin resistance. Rice-husk silica liquid (RHSL) derived from rice husk has the ability to improve the dysfunction of pancreatic β-cells. This study aimed to confirm the potential protective effects of RHSL in streptozotocin (STZ)-induced diabetic mice. Diabetes was induced in male C57BL/6J mice by intraperitoneal administration of STZ (200 mg/kg BW). RHSL, food-grade silica liquid (FDSL), and rosiglitazone (RSG) were administered to diabetic mice for 12 weeks after successful induction of diabetes. During the experiment, fasting blood glucose, serum insulin, and organ weights were measured. The histopathology of liver tissue was evaluated by H&E staining. Western blotting was performed to assess protein expression levels. The results showed that RHSL significantly reversed the serum insulin levels and improved oral glucose tolerance test (OGTT) results (*p* < 0.05). In addition, liver sections of STZ-induced diabetic mice after RHSL treatment showed neatly arranged and intact hepatocytes. Furthermore, RHSL was more effective than FDSL in increasing the expression of SIRT1 and decreasing the expression of the PPAR-γ and p-NF-κB proteins. Taken together, this study demonstrated that RHSL ameliorated STZ-induced insulin resistance and liver tissue damage in C57BL/6J mice.

## 1. Introduction

Type 2 diabetes mellitus (T2DM) is a well-known chronic metabolic disease [1], and diabetic liver injury is one of its complications, which can seriously affect the quality of life of diabetic patients through dysregulation of glucose and lipid metabolism [2,3]. When adipocytes accumulate in the liver due to disorders of lipid metabolism, the liver develops insulin resistance which, in turn, leads to hyperglycemia and glucose metabolism disorders. The mechanisms of dysregulation are intertwined; the induction of hyperglycemia promotes insulin resistance which, in turn, forces the surrounding tissues to utilize glucose in large quantities, resulting in poor glucose tolerance [4]. In addition, this induces a series of inflammatory-response-related pathways through the production of reactive oxygen species (ROS). This continues in a vicious circle and, eventually, the liver suffers more damage.

The construction of T2DM animal models is mainly based on transgenic animals and chemical reagent induction [5], in which streptozotocin (STZ) is often used in animal experiments to simulate diabetes models—depending on the structural similarity of STZ to 2-deoxy-D-glucose—and can selectively destroy pancreatic β-cells via glucose transporter (GLUT)-2 [6,7]. However, since hepatocytes and renal tubular cells also possess GLUT-2 [8], they are highly sensitive to STZ; therefore, hepatic and renal damage are also found in the STZ-induced diabetes model. Recent studies have shown that the STZ-induced hyperglycemia pattern multiplies thioacetamide-induced acute liver injury, possibly through inhibition of the AMP-activated protein kinase (AMPK)/mammalian target of rapamycin (mTOR)-mediated induction of autophagy, resulting in NOD-like receptor family pyrin domain-containing protein 3 (NLRP3) inflammasome activation in Kupffer cells [9].

Several studies have suggested that the mechanism underlying STZ-induced liver injury is related to the inflammatory response, possibly through the induction of ROS, nitric oxide (NO) production, and initiation of downstream nuclear factor-κB (NF-κB) pathways by glucose or free fatty acid overload [10]. Studies have been focusing on molecules that can interfere with the NF-κB pathway and, thus, ameliorate T2DM-induced liver damage [11]. Exploring such key molecules offers prospects for the potential treatment of chronic T2DM [12].

Silicon (Si), a micronutrient with multiple functions in plant growth and human health, is thought to improve rice growth and reduce the toxicity of environmental pollutants such as 1,2,4-trichlorobenzene (TCB) to plants by regulating antioxidant enzyme systems, photosynthesis, and osmotic pressure [13]. Si has been previously reported to exhibit antidiabetic effects via improving insulin resistance and lowering blood glucose levels, thereby reducing the risk of glomerulopathy [14,15]. Our previous study revealed the potential antidiabetic effect of rice-husk silica liquid (RHSL) on pancreatic β-cells by inducing autophagy and attenuating STZ-induced ROS-mediated apoptosis [16]. Although previous studies have shown that rice husks are rich in silica, and that nanosized silica is more biocompatible [17], the information on rice-husk silica (RHS) in food inspection and functional analysis is still limited.

Consequently, the aim of the present study was to explore the ability of RHSL to ameliorate STZ-induced impairment in a diabetic model of C57BL/6J mice, and to further identify the possible mechanisms involved.

## 2. Materials and Methods

### 2.1. Reagent Preparation

#### 2.1.1. Rice-Husk Silica Liquid (RHSL) and Food-Grade Silica Liquid (FDSL)

The detailed process of rice husk preparation can be found in previous studies [18]. In addition, the production process and the conditions of aqueous RHSL (rice-husk silica liquid) were performed according to our previous research [16]. The produced standard solution was used at the original concentration and diluted with double-distilled water (ddH_2_O) to obtain an effective dose, which was used in the subsequent experiments.

Silicon dioxide (SiO_2_; MW: 60.08; CAS number: 60676-86-0; purity ≥ 99%) was purchased from ECHO CHEMICAL Co., Ltd. (Miaoli, Taiwan). Silicon dioxide was diluted with ddH_2_O to obtain the effective dose used for the subsequent experiments; this solvent was defined as food-grade silica liquid (FDSL).

#### 2.1.2. Streptozotocin (STZ)

Streptozotocin (STZ, C_8_H_15_N_3_O_7_; MW: 265.2; CAS number: 18883-66-4; purity ≥ 95%) was obtained from Cayman Chemical (Ann Arbor, MI, USA) as a white-to-light-yellow crystalline solid. STZ was freshly prepared with sodium citrate buffer (pH 4.5) containing 0.1 M Na citrate (Sigma-Aldrich, St. Louis, MO, USA) and 0.1 M citric acid (Sigma-Aldrich, St. Louis, MO, USA) and administered to mice (200 mg/kg body weight) within 30 min for the induction of T2DM.

#### 2.1.3. Rosiglitazone (RSG)

Rosiglitazone (RSG, C_18_H_19_N_3_O_3_S; MW: 357.4; CAS number: 122320-73-4; purity ≥ 98%) was obtained from Cayman Chemical (Ann Arbor, MI, USA), dissolved with dimethyl sulfoxide (DMSO) at the highest concentration of 12.5 mg/mL, and finally diluted with ddH_2_O before administration to mice.

### 2.2. Establishment of Diabetic Mice and Treatment Interventions

Thirty-four 8-week-old male C57BL/6J (B6) mice were purchased from BioLASCO (BioLASCO, Taipei, Taiwan). The mice were individually housed at 20–25 °C in 50 ± 10% relative humidity with a 12-hout light–dark cycle. After the mice were acclimatized for 2 weeks, their blood glucose (fasting for 12 h) was measured in the 0th week of the experiment. After obtaining the basal blood glucose, the control group was given 0.9% saline by intraperitoneal injection, while the induction group (i.e., the rest of the mice) was given STZ (200 mg/kg BW) by intraperitoneal injection, followed by observation of random blood glucose once weekly for 2 weeks. Moreover, the food intake, water intake (average per cage), and individual body weight were recorded. Except for the control group, mice were randomly divided into 6 groups after confirming the induction of diabetes (i.e., blood glucose level over 250 mg/dL). Each group was given different interventions by oral gavage (6 days/week; feeding amount of 200 μL) as follows: control group (ddH_2_O, n = 5), STZ group (ddH_2_O, n = 5), STZ+RHSL 1/100 group (100-fold dilution in ddH_2_O, n = 5), STZ+RHSL 1/400 group (400-fold dilution in ddH_2_O, n = 5), STZ+FDSL 1/100 group (100-fold dilution in ddH_2_O, n = 5), STZ+FDSL 1/400 group (400-fold dilution in ddH_2_O, n = 5), STZ+RSG 5 mg/kg group (n = 4). Food intake, water intake, and body weight were monitored weekly. After 12 weeks, the oral glucose tolerance test (OGTT, 7 time points) was performed and, finally, the mice were euthanatized, their blood was collected, and the relevant organs were harvested for analyses. All animal procedures conducted in this study adhered to a protocol approved by the IACUC (Institutional Animal Care and Use Committee; LAC-2018-0034) of Taipei Medical University.

### 2.3. Determination of Organ Indices

The different organ indices were calculated by dividing the individual organ weight by the whole body weight and then multiplying by 100; the results were statistically analyzed using SigmaPlot, version 12.5 (SoftHome, Taipei, Taiwan).

### 2.4. Oral Glucose Tolerance Test (OGTT)

Mice were fasted overnight, followed by oral gavage of glucose (2 g/kg body weight). Blood samples were collected from the tail vein immediately at 0, 15, 30, 60, 90, 120, and 180 min after gavage. Glucose tolerance was expressed as the area under the curve (AUC) of glucose concentrations between 0 and 180 min. Blood glucose levels were determined using a glucometer (Fora Care Inc., Moorpark, CA, USA).

### 2.5. Plasms Insulin Analysis

Plasma insulin levels were measured using a commercial enzyme-linked immunosorbent assay (ELISA) kit (Mercodia; 10-1247-01, Uppsala, Sweden) in accordance with the instructions of the manufacturer. The absorbance levels for each sample at 450 nm were measured using a microplate reader (BioTek, Winooski, VT, USA).

### 2.6. Assessment of Liver Pathology

Liver tissue samples were immersed in 4% paraformaldehyde; paraffin-embedded tissue was then sectioned and stained with hematoxylin and eosin (H&E), which was entrusted to BIO-CHECK LABORATORIES LTD. (New Taipei City, Taiwan) for processing. Photomicrographs were taken with an EVOS microscope to visualize the damage in the liver sections.

### 2.7. Western Blotting Analysis

Liver samples (about 0.06 g) were digested with 300 μL of lysis buffer (mixed with a protease and phosphatase inhibitor cocktail (Roche, Basel, Switzerland)). Samples containing 30 μg of proteins were subjected to SDS–PAGE gels and transferred to a PVDF membrane (0.45 µm or 0.22 µm, for 100 min at 100 V). The membranes were blocked with 5% bovine serum albumin (BSA; BioShop, Burlington, ON, Canada) for 1 h and then probed with primary antibodies at 4 °C overnight, including anti-PPAR-γ, anti-SIRT, and anti-p-NF-κB, which were purchased from Cell Signal Technology (Beverley, MA, USA). Anti-horseradish peroxidase (HRP)-conjugated glyceraldehyde 3-phosphate dehydrogenase (GAPDH) was purchased from Proteintech (Rosemont, IL, USA). Subsequently, the membranes were incubated with the corresponding goat anti-rabbit antibody IgG (Abcam, Cambridge, UK) for 1 h. The chemiluminescence was imaged using the eBlot Touch Imager^TM^ (eBlot Photoelectric Technology, Shanghai, China) after being reacted with electrochemiluminescence (ECL; Thermo Fisher Scientific, Waltham, MA, USA). Densitometric estimations were quantified using the ImageJ software (National Institutes of Health, NIH, Bethesda, MD, USA). All of the raw data obtained using Western blotting are presented in Supplementary Figure S1.

### 2.8. Statistical Analysis

Data are expressed as the mean ± standard deviation (SD) and were analyzed using SigmaPlot, version 12.5 (SoftHome, Taipei, Taiwan, China). Statistical significance was determined by two-tailed Student’s *t*-tests (for comparison of two groups) and one-way analysis of variance (ANOVA) followed by Tukey’s post hoc analysis (for comparison of three or more groups). The difference between two means was considered statistically significant when *p* < 0.05 or *p* < 0.001.

## 3. Results

### 3.1. Effects of RHSL and FDSL on Body Weight and Organ Weight in STZ-Induced Diabetic Mice

**Figure 1:**
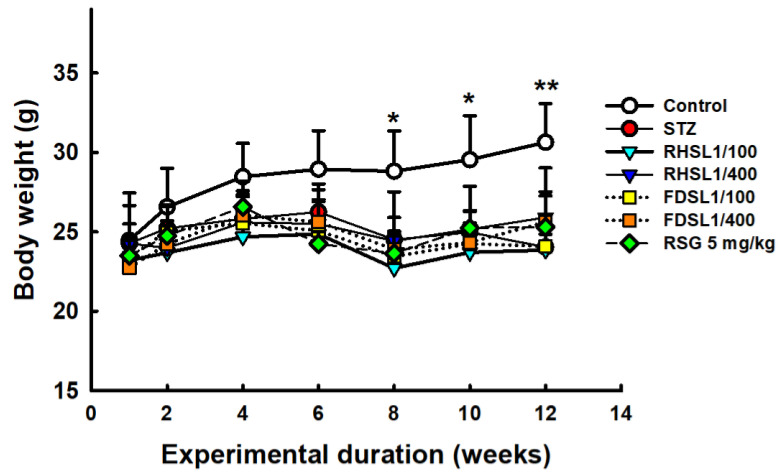
Body weight changes in STZ-induced diabetic mice: Results are expressed as the means ± SD (n = 5 per group), * *p* < 0.05 or ** *p* < 0.001 compared with the STZ alone group. STZ, streptozotocin; RHSL, rice-husk silica liquid; FDSL, food-grade silica liquid; RSG, rosiglitazone.

Weight loss is common in diabetic patients. The results showed that all streptozotocin-injected animals lost significantly more body weight after 8 weeks compared to age-matched controls (Figure 1). However, different treatments had limited effects on improving STZ-induced weight loss compared to the STZ alone group (Figure 1). In addition, we relied on the extent of organ damage as an estimate for the course of the induced diabetes pattern. The results showed that none of the STZ-injected animals showed significantly altered effects in the lungs, spleen, kidneys, or testes compared to age-matched controls, while the liver weights were significantly increased after STZ induction (Table 1). However, liver weight was not reversed in the treated groups compared to the STZ group.

### 3.2. Effects of RHSL and FDSL on Water Intake, Food Intake, and Blood Glucose in STZ-Induced Diabetic Mice

The induction of the diabetic mouse model was observed through monitoring the mice’s dietary behavior. Table 2 shows that the water intake of the STZ group was significantly increased compared to the control group (*p* < 0.05). Interestingly, the water intake of the high-dose RHSL group was significantly decreased compared with the STZ group (*p* < 0.05). In addition, RSG (5 mg/kg) was also found to significantly reduce the water intake of STZ-induced mice in the positive control group (*p* < 0.05). However, in terms of food intake, there was no significant difference in the increase in food intake between the STZ group and the control group, and the other groups only had a downward trend compared with the STZ group (Table 2).

**Figure 2:**
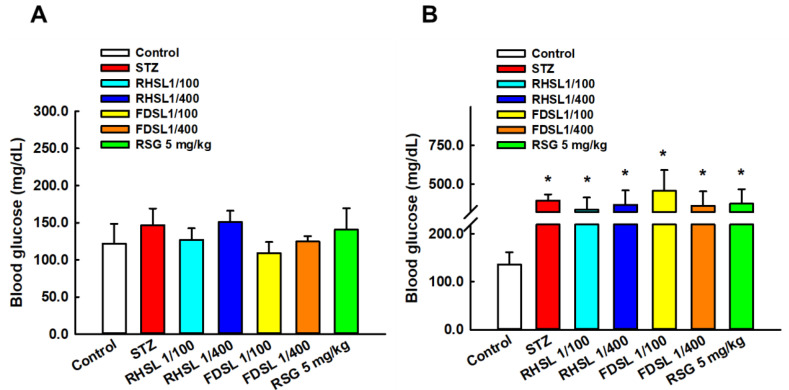
Changes in blood glucose before and after STZ intervention in diabetic mice: Changes in blood glucose in each group (**A**) before STZ intervention and (**B**) after STZ intervention. Results are expressed as the means ± SD (n = 5 per group); * *p* < 0.05 compared with the control group. STZ, streptozotocin; RHSL, rice-husk silica liquid; FDSL, food-grade silica liquid; RSG, rosiglitazone.

Fasting blood glucose level is an important indicator for establishing diabetic mouse models. Figure 2A shows the values of basal fasting blood glucose before induction, which were not significantly different between groups. Figure 2B shows that the blood glucose values of all STZ mice were higher than 250 mg/dL after induction with STZ (200 mg/kg), indicating that the diabetic mouse model was successfully induced.

### 3.3. Effects of RHSL and FDSL on Blood Glucose Regulation and Insulin Tolerance in STZ-Induced Diabetic Mice

**Figure 3:**
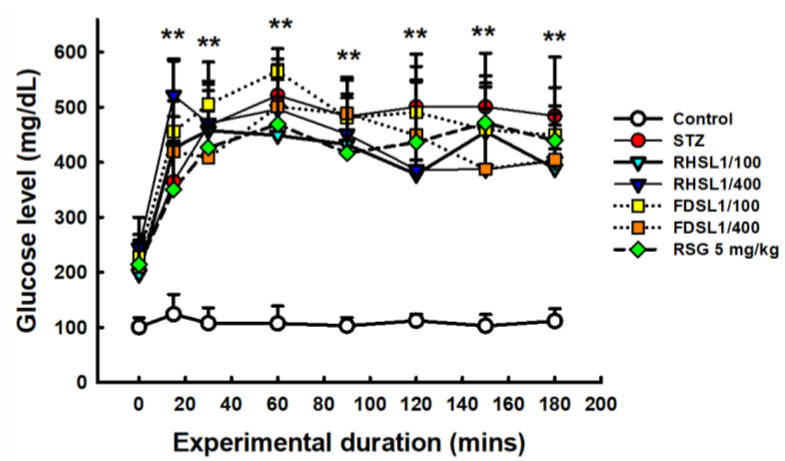
Changes in serum glucose in response to oral glucose loading in STZ-induced diabetic mice: Blood was collected at 0, 15, 30, 60, 120, 150, and 180 min after glucose loading (2 g/kg) for determination of serum glucose. Results are expressed as the means ± SD (n = 5 per group); ** *p* < 0.001 compared with the control group. STZ, streptozotocin; RHSL, rice-husk silica liquid; FDSL, food-grade silica liquid; RSG, rosiglitazone.

To validate that the insulin-resistant mouse model was successfully established, OGTT and insulin levels in the mice were assessed. As shown in Figure 3, the OGTT curves of STZ or STZ combined with RHSL or FDSL and the RSG group were significantly higher than those of the control group (*p* < 0.05), but there was no significant difference between the groups after STZ induction, which we attribute to individual differences, since the error bars were found to be too large. However, after calculating the AUC, we found that the AUC value of the high-dose RHSL group was significantly lower than that of the STZ group, and the RSG group also showed the same result, where its AUC value was significantly lower than that of the STZ group (Table 3).

**Figure 4:**
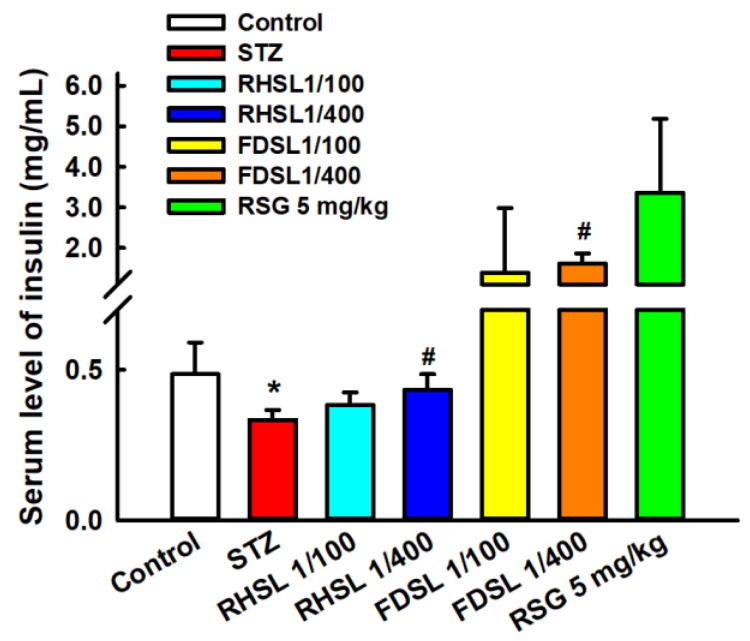
Changes in insulin concentration in STZ-induced diabetic mice: Results are expressed as the means ± SD (n = 5 per group); * *p* < 0.05 compared with the control group; # *p* < 0.05 compared with the STZ group. STZ, streptozotocin; RHSL, rice-husk silica liquid; FDSL, food-grade silica liquid; RSG, rosiglitazone.

Furthermore, the insulin sensitivity results in Figure 4 show that the STZ group had significantly lower serum insulin concentrations compared to the control group (*p* < 0.05), and that the low-dose RHSL group and low-dose FDSL group were found to significantly reverse the STZ-induced low insulin concentration (*p* < 0.05); however, there were no significant differences between the high-dose RHSL group and the high-dose FDSL group compared with the STZ group. These results reveal that the mice showed significant improvement in insulin secretion after the treatment intervention.

### 3.4. Effects of RHSL and FDSL on Liver Morphology in STZ-Induced Diabetic Mice

**Figure 5:**
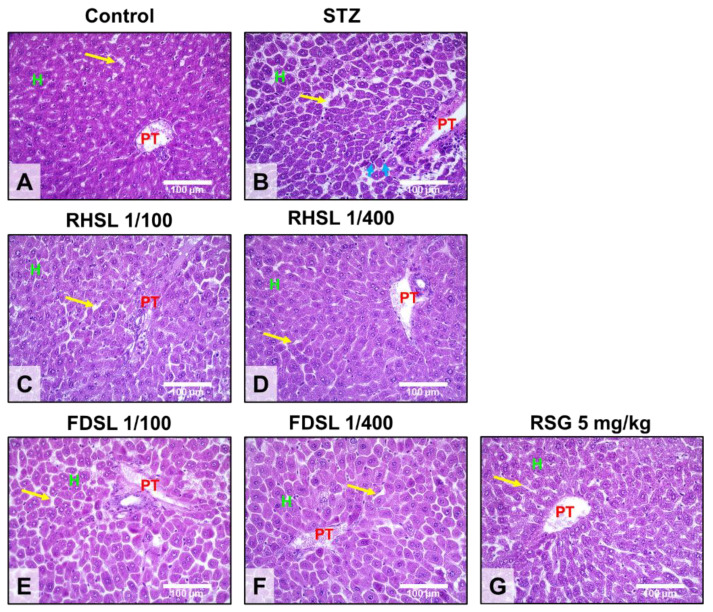
Effects of RHSL and FDSL treatment on the liver phenotype in STZ-induced diabetic mice. Representative images of hepatic hematoxylin and eosin staining showing the locations of the portal triad (PT), hepatocytes (H), sinusoidal spaces (yellow arrows), and leukocytic infiltration (blue arrows) in (**A**) normal control and (**B**) STZ-induced diabetic mice treated with (**C**,**D**) RHSL, (**E**,**F**) FDSL, and (**G**) RSG. Magnifications, ×400; scale bar = 100 μm. STZ, streptozotocin; RHSL, rice-husk silica liquid; FDSL, food-grade silica liquid; RSG, rosiglitazone.

Liver sections from control mice showed normal liver architecture, and it was observed using H&E staining that the liver consisted of a central vein (CV), a portal triad (i.e., portal vein, hepatic artery, and bile duct), and an anastomotic network of healthy hepatocytes (H) arranged in strands, some of which showed binucleation due to regeneration (Figure 5A), whereas liver sections of STZ-induced diabetic mice showed severe pathological changes, as can be seen from the staining pattern, portal vein dilatation and hyperemia in the liver, severe cytoplasmic degeneration of hepatocytes, and wall destruction, which caused sinusoidal spaces (yellow arrows) to expand along with the appearance of significant leukocyte infiltration (blue arrows) (Figure 5B). On the other hand, our results showed that, compared to the FDSL group (Figure 5E,F), the RHSL group (Figure 5C,D) found better improvement in the livers of STZ-induced diabetic mice—not only was the hepatic sinusoidal space narrowed, but also the arrangement and integrity of the hepatocytes tended to recover. In addition, the livers of STZ-induced diabetic mice also tended to be reversed after RSG treatment (Figure 5G).

### 3.5. Effects of RHSL and FDSL on Hepatic Glucose-Regulation-Related Markers in STZ-Induced Diabetic Mice

**Figure 6:**
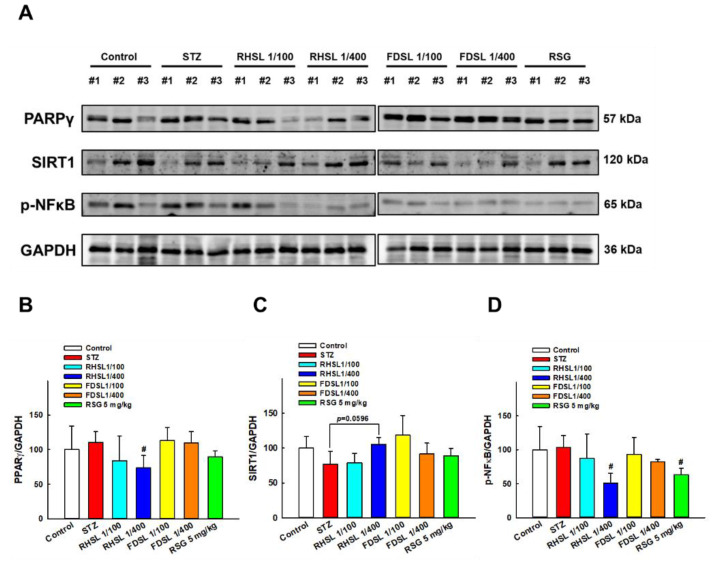
Effects of RHSL supplementation on hepatic protein levels of glucose- or energy-metabolism-related markers in STZ-induced diabetic mice: (**A**) Representative images of Western blotting analysis of hepatic PPAR-γ, SIRT1 and p-NF-κB expression. Densitometric quantification of (**B**) PPAR-γ, (**C**) SIRT1, and (**D**) p-NF-κB expression normalized to GAPDH. Results are expressed as the means ± SD (n = 5 per group); # *p* < 0.05 compared with the STZ group. STZ, streptozotocin; RHSL, rice-husk silica liquid; FDSL, food-grade silica liquid; RSG, rosiglitazone.

As shown in Figure 6A,B, compared with the control group, the expression of PPAR-γ after STZ induction only increased, but did not reach a significant difference, while the PPAR-γ level in the low-dose RHSL group was significantly lower than that in the STZ group (*p* < 0.05), meaning that RHSL has the potential to improve liver function and blood sugar regulation. On the other hand, as shown in Figure 6A,C, the expression of the SIRT1 protein in the low-dose RHSL group was higher than that in the STZ group (*p* = 0.0596), while the expression of the p-NF-κB protein in the low-dose RHSL group and RSG group was significantly lower than that in the STZ group (*p* < 0.05, Figure 6A,D). These results show that RHSL has the potential to ameliorate liver inflammation in STZ-induced diabetic mice. However, there were no significant differences between the other treatment groups.

## 4. Discussion

**Figure 7:**
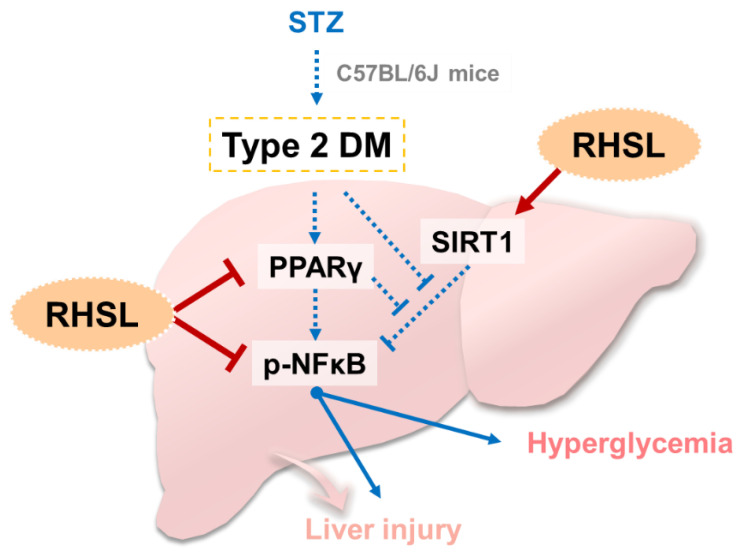
**Summary and hypothesis of mechanisms of RHSL-mediated therapeutic effects in STZ-induced diabetic mice:** STZ, streptozotocin; RHSL, rice-husk silica liquid; PPAR-γ, peroxisome proliferator-activated receptor gamma; NF-κB, nuclear factor kappa B; SIRT1, sirtuin 1.

The present study validates the potential efficacy of rice-husk silica liquid (RHSL) against diabetes in an animal model of STZ-induced T2DM. We found that this plant-derived silicon not only restored serum insulin concentrations and improved glucose tolerance, but also ameliorated liver tissue damage and reversed the expression of glucose-metabolism-related markers in a STZ-induced diabetic mouse model. The proposed mechanisms of RHSL’s action in the therapy of STZ-induced diabetic mice are summarized in Figure 7.

Streptozotocin (STZ) is often used alone or in combination with other dietary patterns as an effective way to induce T2DM in animals [19]. In a recent study using STZ at 200 mg/kg BW to induce diabetes in mice, the authors found that blood glucose levels in the induced group were elevated until the third week and remained at around 400 mg/dL [20]. Our experiments used the same dose and found that 200 mg/kg STZ induced blood glucose levels that were also maintained above 400 mg/dL, indicating that the induction pattern was successfully established. Hyperglycemia is often associated with a variety of diseases, and common symptoms include polydipsia, polyuria, polyphagia, weight loss, and decreased ability to fight infection [21]. In the present study, we also observed the pathophysiological state of mice after STZ intervention and found that STZ-induced mice showed a trend towards weight loss and significantly increased water intake compared to controls. Unfortunately, there were no significant differences in food intake between groups. The regulation of blood glucose depends on the release of insulin. In our previous study, we showed that RHSL abrogated the effect of streptozotocin (STZ) on the mass of RIN-m5F pancreatic β-cells and their ability to secrete insulin [16]. In the present study, we observed similar results, where RHSL restored blood insulin concentrations, meaning that RHSL has the potential to reverse the STZ-induced insulin resistance in C57BL/J6 mice.

Based on the previous findings detailing the liver morphology in STZ-induced diabetes [22], we also stained the liver tissues of each group in the present study for morphological analysis. The pattern of STZ-induced liver injury usually shows severe pathological changes, including portal vein dilatation and congestion, wall destruction, surrounding leukocyte infiltration, and even severe cytoplasmic degeneration of hepatocytes [22]. Previous studies have shown mildly dilatated hyperemic central veins and marked hepatocyte microvesicular steatosis in liver sections of the untreated diabetes group, whereas administration of melatonin reduced inflammation and hepatocyte alignment in the liver sections [23]. Similar histological staining results were also obtained in the present study, indicating that STZ-induced liver tissue damage can be confirmed by observing the incomplete arrangement of liver cells, abnormal morphology of the portal triad (consisting of the portal vein, hepatic artery, and bile duct), loose liver tissue, widening of the hepatic sinusoidal space, and even the presence of infiltrating immune cells. However, compared with the STZ alone group, the staining of the liver tissue in the RHSL group showed a more complete arrangement of hepatocytes, a narrowed hepatic sinusoidal space, and a more normal morphology of the portal triad. Collectively, RHSL has the potential to reverse STZ-induced liver damage in C57BL/J6 mice.

Peroxisome proliferator-activated receptor gamma (PPAR-γ) is mainly involved in lipid and glucose metabolism, and its activation is thought to promote insulin resistance [24]. In addition, previous studies have shown that PPAR-γ promotes hepatic inflammation by exacerbating hepatic lipid accumulation [25,26]. In a diabetes model induced by a high-fat diet (four weeks) combined with STZ injection (40 mg/kg, single dose), it was found that the expression of PPAR-α was lower and the expression of PPAR-γ was higher in diabetic rats, but the intervention with *Epigynum auritum* extract not only increased the expression of PPAR-α but also inhibited the expression of PPAR-γ in the liver of diabetic rats [27]. Similarly, in the diabetes model induced by a high-fat diet (four weeks) combined with STZ injection (40 mg/kg, 2 times), it was found that the expression of PPAR-α was lower and the expression of PPAR-γ was higher in diabetic mice, but the intervention with a flavonoid-rich extract from *Sophora alopecuroides* L. not only increased the expression of PPAR-α but also inhibited the expression of PPAR-γ in the livers of diabetic mice [28]. However, a study with multiple low-dose STZ injections (60 mg/kg, 4 times) after being fed with a high-fat diet (six weeks) showed different results, finding that PPAR-γ protein levels were significantly reduced in the livers of T2DM mice, while *Angelica sinensis* polysaccharide treatment significantly increased PPAR-γ expression [29]. The results of the present study were similar to those of previous studies; we found that PPAR-γ expression in the liver had a trend of increasing after STZ induction, but the PPAR-γ expression in the liver was significantly decreased after administration of RHSL.

Sirtuin 1 (SIRT1) is a nicotinamide adenine dinucleotide (NAD (+))-dependent protein deacetylase that is reported to be involved in numerous important biological processes, including improved glucose homeostasis and insulin sensitivity [30]. In a rat model of T2DM, a SIRT1 agonist (SRT1720) was shown to ameliorate the effects of diabetes-induced cognitive decline [31]. Previous studies have shown that N1-methylnicotinamide (MNAM) regulates insulin sensitivity and hepatocyte gluconeogenesis in mice with type 2 diabetes by activating SIRT1 and inhibiting the acetylation of FOXO1 [32]. A previous study exploring the relationship between PPAR-γ and SIRT1 showed that stimulation of PPAR-γ mRNA expression can modulate *SIRT1* mRNA expression [33]. In the diabetes model induced by a low-dose STZ injection (30 mg/kg) combined with a high-fat diet, the results of Western blotting showed that PPAR was significantly increased, whereas SIRT1 was significantly decreased, in the liver [34]. In the present study, we observed similar results, in that SIRT1 expression in the liver had a trend of decreasing after STZ induction, but the SIRT1 expression in the liver was significantly increased after the administration of RHSL.

Nuclear factor-κB (NF-κB) is a central mediator in the regulation of innate and adaptive immune cell function and plays a pivotal role in the expression of genes mainly involved in inflammation [35,36]. Numerous studies have shown that the expression of NF-κB is upregulated in the liver after the induction of diabetes, indicating that inflammation-related pathways are involved in STZ-induced diabetes [37,38]. A recent review has also determined that some antidiabetic drugs used in the treatment of liver fibrosis are also involved in downregulating the expression of NF-κB [3]. Moreover, there is emerging evidence indicating that SIRT1 deacetylates NF-κB [39]. A previous study showed that betaine significantly upregulated SIRT1 and downregulated *NF-κB* mRNA expression compared to diabetic control rats [40]. In the present study, our results showed that the expression of NF-κB in the liver was significantly reduced after the administration of RHSL in a STZ-induced diabetic mice model, suggesting that RHSL may have the potential to reduce inflammation.

The RHSL and FDSL used in this study were formulated according to the silicon concentration, and the high dose contained about 100 ppm of silicon, which is within the safe range. Our previous study showed that zebrafish embryos and juveniles were not affected in terms of survival, hatching, or deformity rates when exposed to less than 200 µg/mL RHS and displayed a healthy physiological state [18]. Silicon dioxide is an atomic crystal that is insoluble in water and requires a strong alkali to dissolve. In this study, RHSL derived from rice husks was fired and dissolved with alkali and then diluted with water to the original concentration. Likewise, FDSL from commercial food-grade silica powder, dissolved in alkali and then diluted with water, was used as a control sample for RHSL. Therefore, the difference between the two interventions lies in the source, and their silicon content in the original solution was the same (close to 10,000 ppm).

The limitations of this article include the following: First, the use of RHSL for the treatment of diabetes is novel, but the benefits were less explored in the present study, and there is little evidence to support its efficacy. Second, this study used a high-dose STZ-induced diabetes model, which is different from other induced models with a low dose of STZ combined with a high-fat diet, which may be the reason for the poor hypoglycemic effect of the intervention. Finally, in the present study, we found most of the data to support lower doses being more effective than higher doses, and we speculate that there may be two reasons involved: First, the intragroup differences were so large that there was no difference between the high-dose group and the induced group. Second, the solubility and absorption rate of silicon were saturated, and the silicon could not be used effectively, so the effect of the high-dose group was not as good as expected.

## 5. Conclusions

In this study, we found that RHSL reverses insulin secretion and increases the sensitivity of peripheral tissue to insulin, thereby improving glucose tolerance. In addition, RHSL ameliorates STZ-induced liver damage by reversing PPAR-γ/SIRT1/p-NF-κB expression. Overall, RHSL has the potential to improve the STZ-induced diabetic mice model, and this study provides the basis for the application of RHSL to clinical trials in the future.

## Figures and Tables

**Table 1:** Weight of different organs.

	Lung	Liver	Spleen	Kidney	Testis
Group	Relative Organ Weight (*w*/*w*, %)				
Control	0.61 ± 0.14 ^a^	4.14 ± 0.43 ^a^	0.20 ± 0.08 ^a^	1.34 ± 0.12 ^a^	0.70 ± 0.13 ^a^
STZ	0.78 ± 0.15 ^a^	5.66 ± 0.44 ^b^	0.23 ± 0.04 ^a^	1.33 ± 0.11 ^a^	0.79 ± 0.12 ^a^
RHSL 1/100	0.85 ± 0.16 ^a^	5.35 ± 0.22 ^b^	0.19 ± 0.03 ^a^	1.46 ± 0.18 ^a^	0.78 ± 0.14 ^a^
RHSL 1/400	0.69 ± 0.11 ^a^	5.54 ± 1.12 ^b^	0.19 ± 0.01 ^a^	1.57 ± 0.14 ^a^	0.74 ± 0.17 ^a^
FDSL 1/100	0.79 ± 0.08 ^a^	5.91 ± 0.93 ^b^	0.20 ± 0.03 ^a^	1.53 ± 0.33 ^a^	0.82 ± 0.07 ^a^
FDSL 1/400	0.78 ± 0.17 ^a^	5.47 ± 0.35 ^b^	0.23 ± 0.03 ^a^	1.32 ± 0.27 ^a^	0.80 ± 0.12 ^a^
RSG 5 mg/kg	0.78 ± 0.14 ^a^	5.57 ± 0.57 ^b^	0.22 ± 0.06 ^a^	1.40 ± 0.22 ^a^	0.79 ± 0.12 ^a^

STZ, streptozotocin; RHSL, rice-husk silica liquid; FDSL, food-grade silica liquid; RSG, rosiglitazone. Data are presented as the mean ± SD. Values the in same column with different superscript letters indicate significant differences (*p* < 0.05).

**Table 2:** Average of water and food intake (7 days).

Group	Water Intake (mL/Day)	Food Intake (g/Day)
Control	6.03 ± 1.65 ^a^	3.36 ± 0.36 ^a^
STZ	13.94 ± 4.02 ^b^	4.18 ± 0.38 ^a^
RHSL 1/100	9.21 ± 1.49 ^c^	3.97 ± 0.63 ^a^
RHSL 1/400	11.61 ± 1.95 ^b^	3.88 ± 0.48 ^a^
FDSL 1/100	11.40 ± 2.44 ^b^	3.78 ± 0.35 ^a^
FDSL 1/400	10.99 ± 1.86 ^b^	4.05 ± 0.84 ^a^
RSG 5 mg/kg	8.40 ± 1.51 ^c^	4.30 ± 1.11 ^a^

STZ, streptozotocin; RHSL, rice-husk silica liquid; FDSL, food-grade silica liquid; RSG, rosiglitazone. Data are presented as the mean ± SD. Values in the same column with different superscript letters indicate significant differences (*p* < 0.05).

**Table 3:** AUC of blood glucose in the OGTT assay.

Group	AUC of Blood Glucose in the OGTT Assay
Control	12,616.25 ± 239.97 ^a^
STZ	49,509.87 ± 386.14 ^b^
RHSL 1/100	42,681.25 ± 665.69 ^c^
RHSL 1/400	47,890.31 ± 430.31 ^b^
FDSL 1/100	45,690.75 ± 615.60 ^b^
FDSL 1/400	43,638.00 ± 546.32 ^b^
RSG 5 mg/kg	37,191.00 ± 1076.89 ^c^

STZ, streptozotocin; RHSL, rice-husk silica liquid; FDSL, food-grade silica liquid; RSG, rosiglitazone; AUC, area under the curve; OGTT, oral glucose tolerance test. Data are presented as the mean ± SD. Values in the same column with different superscript letters indicate significant differences (*p* < 0.05).

## Data Availability

The data presented in this study are available upon request from the corresponding author. The data are not publicly available due to patent application.

## Supplementary Materials

The following supporting information can be downloaded at: https://www.mdpi.com/article/10.3390/metabo12100964/s1, All of the raw data using Western blotting are presented in Supplementary Figure S1. Raw data of western blotting.
