# Supplementary material for: Effects of Rice-Husk Silica Liquid in Streptozotocin-Induced Diabetic Mice
Source: Metabolites. 2022 Oct 12;12(10):964. doi: 10.3390/metabo12100964 (PMC9611213; doi:10.3390/metabo12100964)
Supplement: Supplementary file 1 [file metabolites-12-00964-s001.zip › metabolites-1956148-supplementary.pdf]

Protective Effects of Rice Husk Silica Liquid in Streptozotocin-Induced Diabetic Mice

-- Supplementary figures

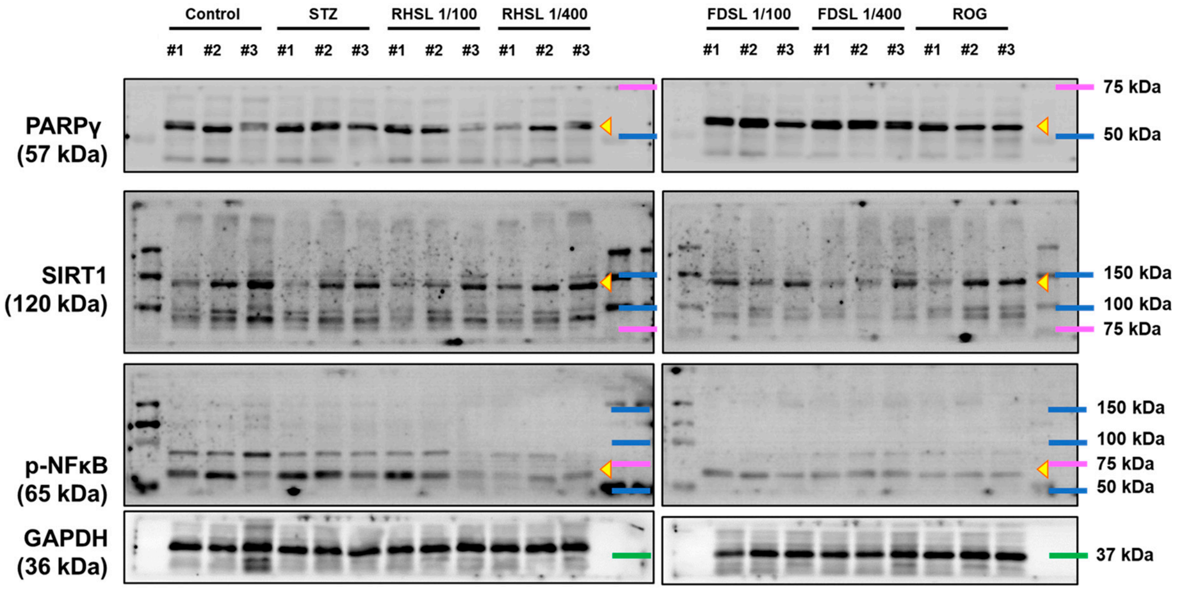

Supplementary Figure S1. Raw data of western blotting.
